# Dr. Oscar Bloch on Removal of Ovaries

**Published:** 1891-12-12

**Authors:** 


### GYNÆCOLOGY.

### DR. OSCAR BLOCH ON REMOVAL OF OVARIES.

Dr. Oscar Bloch, of the Frederick Hospital, Copenhagen, has just added another to the list of oophorectomy operation cases, as described in the *Nord. Med. Arkiv*, Bd. xxiii. n. : r 2. This operation, as is well known, involves the removal of both ovaries with the knife. Previously there had been 11 cases on record; and the interest of this case of Birgitté Kirstine Svendsen, a domestic servant, 30 years of age, depends not only upon the fact that the result was entirely successful in relieving a series of agonising and almost unbearable symptoms, but also because it offers an opportunity of bringing into review the entire series of these cases. This poor woman first experienced painful sensations at the age of 16 years, that is 14 years before she passed under the treatment of Doctor Bloch. Pains in the abdomen, back, breasts, armpits, and headache, with at length violent vomiting of blood and violent tenesmus, rendered life so unendurable that she was willing to undergo any treatment for the sake of relief and to enable her to work again, even at the risk of life. The operation is carefully detailed. It was quite successful, and at an interval of six months' time the patient "was cheerful, complained of no pain, could follow her occupation, and betrayed no signs outwardly that she had lost her ovaries." Passing then in review the 12 cases, our author points out that two died from peritonitis and 10 recovered. Of the 10, six were completely cured, two derived no benefit, got worse rather, while of two no satisfactory history could be obtained. One of the two cases which was not benefited—Hempel's case—offered the peculiarity that a right ovary could not be found, and that some detached portions of the left ovary remained behind after the operation. Granting the validity of this explanation it would appear that of seven cases whose history can be followed, no less than six were found to have been thoroughly relieved. After a careful review of all the facts the author comes to the conclusion that where—and this is generally owing to defective development of the generative system—the patient is rendered unfit for work taken in the widest sense, owing to pain or other circumstances standing in relation to diseased ovulation and all means of alleviation have been found useless the operation for castration is indicated. This suggestive paper is to be commended as well to the notice of surgical specialists as to that of medical men dealing with innumerable forms of abdominal pain more or less connected with the functions of ovulation and menstruation.

